# The Inhibitory Effect of Tartary Buckwheat Extracts on Adipogenesis and Inflammatory Response

**DOI:** 10.3390/molecules22071160

**Published:** 2017-07-12

**Authors:** Mak-Soon Lee, Yoonjin Shin, Sunyoon Jung, Seog-Young Kim, Young-Hee Jo, Chong-Tai Kim, Min-Kyu Yun, Sung-Jin Lee, Johann Sohn, Heui-Jong Yu, Yangha Kim

**Affiliations:** 1 Department of Nutritional Science and Food Management, Ewha Womans University, 52 Ewhayeodae-gil, Seodaemun-gu, Seoul 03760, Korea; troph@hanmail.net (M.-S.L.); yjin19@hotmail.com (Y.S.); cococosy@naver.com (S.J.); saraha9390@gmail.com (S.-Y.K.); 2 Research Group of Bioprocess Engineering, Korea Food Research Institute, Seongnam, Gyeonggi 13539, Korea; cho6452@naver.com (Y.-H.J.); ctkim@kfri.re.kr (C.-T.K.); 3 R&D Center, SKBioland Co. Ltd., 152, Manhae-ro, Danwon-gu, Ansan-si, Gyeonggi-do 15407, Korea; minkyu@skbioland.com (M.-K.Y.); sungjinreal@skbioland.com (S.-J.L.); jhsohn@skbioland.com (J.S.); hjyu@skbioland.com (H.-J.Y.)

**Keywords:** tartary buckwheat, adipogenesis, inflammatory response, 3T3-L1 adipocytes

## Abstract

Tartary buckwheat (*Fagopyrum tataricum*) has been established globally as a nutritionally important food item, particularly owing to high levels of bioactive compounds such as rutin. This study investigated the effect of tartary buckwheat extracts (TBEs) on adipogenesis and inflammatory response in 3T3-L1 cells. TBEs inhibited lipid accumulation, triglyceride content, and glycerol-3-phosphate dehydrogenase (GPDH) activity during adipocyte differentiation of 3T3 L1 cells. The mRNA levels of genes involved in fatty acid synthesis, such as peroxisome proliferator-activated receptor-γ (PPAR-γ), CCAAT/enhancer binding protein-α (CEBP-α), adipocyte protein 2 (aP2), acetyl-CoA carboxylase (ACC), fatty acid synthase (FAS), and stearoylcoenzyme A desaturase-1 (SCD-1), were suppressed by TBEs. They also reduced the mRNA levels of inflammatory mediators such as tumor necrosis factor-α (TNF-α), interleukin-6 (IL-6), monocyte chemoattractant protein 1 (MCP-1), and inducible nitric oxide synthase (iNOS). In addition, TBEs were decreased nitric oxide (NO) production. These results suggest that TBEs may inhibit adipogenesis and inflammatory response; therefore, they seem to be beneficial as a food ingredient to prevent obesity-associated inflammation.

## 1. Introduction

Adipocytes play a central role in maintaining whole-body energy balance and lipid homeostasis by storing triglycerides (TGs) or releasing free fatty acids [1,2]. However, excessive fat deposition in adipose tissues can lead to obesity [3]. Obesity is notably associated with various metabolic disorders including hypertension, type 2 diabetes, and cardiovascular diseases [4]. Adipose tissue not only stores fat but is also an important endocrine organ that secretes several physiologically active peptides, including inflammatory cytokines [5]. Obesity causes increased expression of inflammatory cytokines in chronic low-grade inflammation, contributing to pathological dysfunction [6]. Therefore, it is important to develop natural products that might inhibit adipogenesis and inflammation in order to prevent obesity.

Buckwheat (*Fagopyrum esculentum*) is recognized as a functional food and an important source of high quality protein, abundant flavonoids, and well-balanced essential amino acids and minerals [7,8]. Two types of buckwheat, namely common buckwheat (*F. esculentum*) and tartary buckwheat (*F. tataricum*), are consumed globally. Common buckwheat is mainly grown in Europe, USA, Canada, Brazil, South Africa, and Asia. Tartary buckwheat is mainly grown in southwest China, northern India, Bhutan, and Nepal [9,10]. In particular, tartary buckwheat is known to have 9–300 times higher flavonoid content, mainly rutin and quercetin, than common buckwheat [11,12]. In recent years, tartary buckwheat has been attracting research interest for the prevention of various diseases [13,14,15]. Tartary buckwheat is reported to have various beneficial effects, such as antioxidant [16], antitumor [17], and hypoglycemic [18] properties. A recent study reported that rutin-rich tartary buckwheat showed potential effects on decreasing body weight, body fat percentage, and oxidative stress in adult subjects [13]. Yoon and others [19] observed that an 80% ethanolic extract of tartary buckwheat suppressed adipogenesis and reactive oxygen species (ROS) production compared to common buckwheat extract. However, the mechanism of tartary buckwheat extract involved in adipogenesis and inflammatory response remains unclear.

Here, we elucidated the effect of tartary buckwheat extracts (TBEs) on adipogenesis and inflammatory responses and identified the molecular mechanisms underlying its regulatory action. In addition, we investigated the effect of 50% (TBE-50) or 70% (TBE-70) ethanolic extracts of tartary buckwheat on adipogenesis and inflammatory response during adipocyte differentiation.

## 2. Results and Discussion

### 2.1. Rutin Contents of TBE

**Figure 1:**
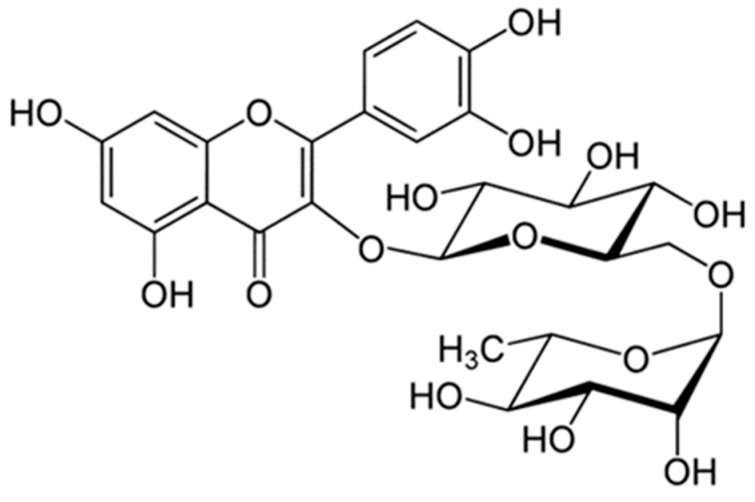
Chemical structure of rutin.

**Figure 2:**
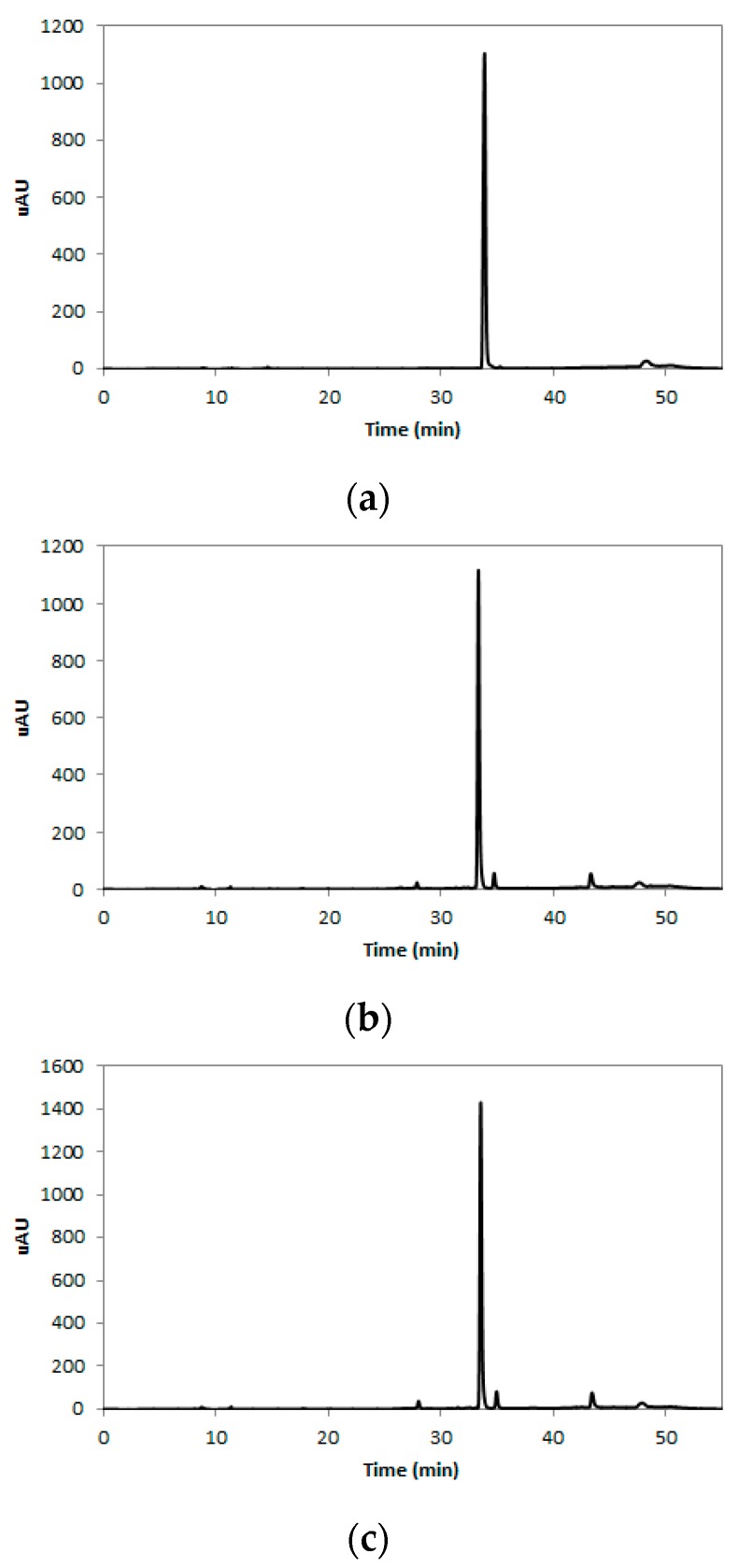
HPLC chromatogram of rutin in TBEs. Rutin standard (**a**); TBE-50 (**b**); TBE-70 (**c**).

**Figure 3:**
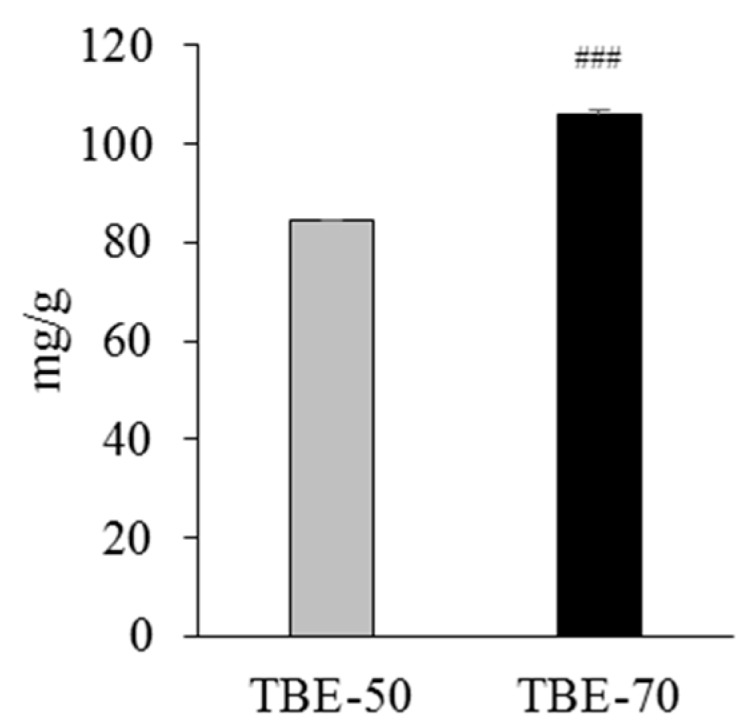
The rutin content of TBE-50 and TBE-70. Rutin content was determined by HPLC. Values are expressed as mean ± SE (*n* = 3) of three independent experiments. ^###^ *p* < 0.001 vs. TBE-50.

Rutin is a flavonol glycoside composed of quercetin and disaccharide rutinose (Figure 1) and is present in many plants, including buckwheat [20]. The tartary buckwheat contains protocatechuic acid, vanilic acid, syringic acid, ferulic acid, sinapic acid, and quercetin as their phenolic compounds [21]. However, many researchers have reported that rutin is the main active compound found in tartary buckwheat extracts, since more than 80% of total flavonoids in tartary buckwheat extracts are revealed to be rutin [21,22], and rutin accounted for about 85–90% of the total anti-oxidative activity in tartary buckwheat [23]. In this study, we analyzed the content of rutin in 50% (TBE-50) or 70% (TBE-70) ethanolic extracts of tartary buckwheat by the high-performance liquid chromatography (HPLC) method. The HPLC chromatogram of the rutin is presented in Figure 2a–c. The rutin concentrations of TBE-50 and TBE-70 were 84.3 ± 0.7 mg/g and 106.0 ± 1.3 mg/g, respectively (Figure 3). Beneficial effects of rutin on obesity [24,25] and inflammation [26,27] have been reported. Thus, we assumed that TBEs might have effects on adipogenesis and inflammation during adipocyte differentiation, and rutin may contribute in part to beneficial effects of TBEs.

### 2.2. Effect of TBEs on 3T3-L1 Cell Viability

**Figure 4:**
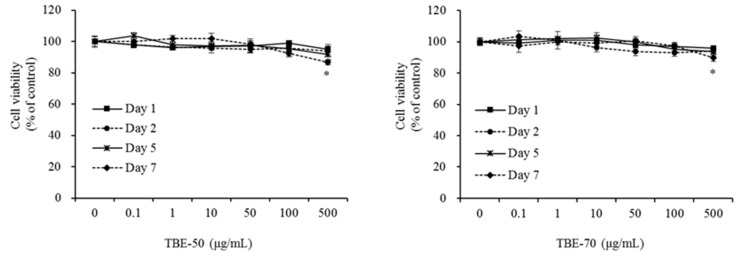
Effects of TBEs on cell viability in 3T3-L1 cells. Cells were treated with 0 (control), 0.1, 1, 10, 50, 100, or 500 µg/mL of TBE-50 (**a**) or TBE-70 (**b**), and incubated for 1, 2, 5, or 7 days. Cell viability was determined using the WST-8 assay. Values are expressed as mean ± SE (*n* = 3) of three independent experiments. * *p* < 0.05 vs. control.

TBEs were tested for the potential cytotoxic effects that might exert on 3T3-L1 cells. Cells were treated with TBE-50 or TBE-70 at various concentrations (0 (control), 0.1, 1, 10, 50, 100, or 500 μg/mL), and incubated for 1, 2, 5, or 7 days. Cytotoxicity was unaffected by 0, 0.1, 1, 10, 50, and 100 μg/mL of TBE-50 and TBE-70 after 7 days of incubation (Figure 4). However, 7 days of incubation with high doses (500 μg/mL) of TBE-50 and TBE-70 significantly decreased cell viability by 13.2% and 10.3%, respectively, compared to the control. The cells did not show any toxicity upon treatment with TBE-50 and TBE-70 at concentrations of 0.1–100 µg/mL. Therefore, this in vitro study was performed at a nontoxic range of concentrations below 100 μg/mL with both extracts.

### 2.3. Effects of TBEs on Lipid Accumulation and TG Content during Adipocyte Differentiation

**Figure 5:**
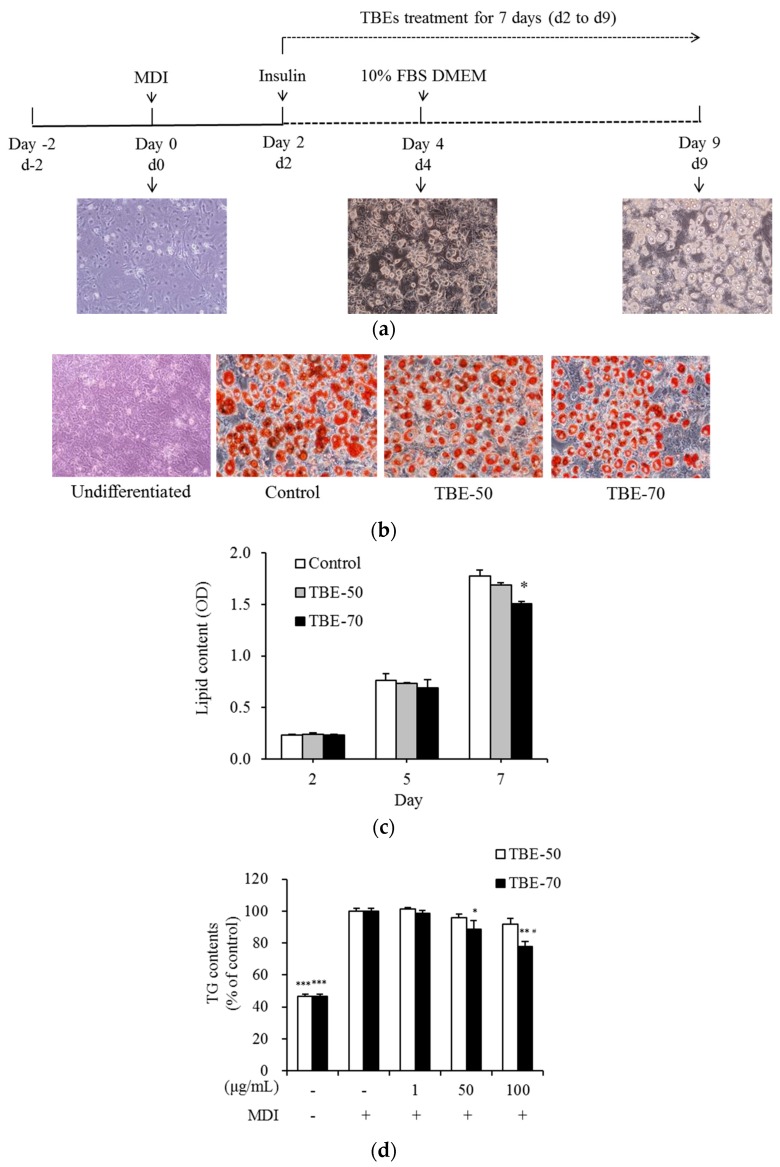
Effects of TBEs on intracellular lipid accumulation and TG content during adipocyte differentiation. Scheme of the 3T3-L1 preadipocyte differentiation experiment (**a**). 3T3-L1 cells were treated with 100 µg/mL of TBE-50 or TBE-70, and incubated for 2, 5, or 7 days (d2 to d9). On Day 7 (d9), change of adipocyte differentiation was presented with Oil Red O staining (**b**). Intracellular lipid content (**c**) was stained with oil-red O dye, and dissolved the stained oil droplets with isopropanol and quantified by spectrophotometric analysis. Representative cell images were captured at 200× magnification. 3T3-L1 cells were treated with 0 (MDI treated control), 1, 50, and 100 µg/mL of TBE-50 and TBE-70, and incubated for 7 days. Intracellular TG content (**d**) was determined using enzymatic colorimetric methods. MDI, medium containing 3-isobutyl-1-methylxanthine, dexamethasone and insulin. Values are expressed as mean ± SE (*n* = 3) of three independent experiments. * *p* < 0.05, ** *p* < 0.01 and *** *p* < 0.01 vs. MDI-treated control. ^#^ *p* < 0.05 vs. TBE-50.

Adipogenesis is the process by which preadipocytes become mature adipocytes, when exposed to appropriate environmental condition and gene expression. Mature adipocytes enlarge in size while accumulating lipids that finally fill the cells. In this study, the effects of TBEs on intracellular lipid accumulation and triglyceride (TG) content during 3T3-L1 adipocyte differentiation were measured. Cells were treated with 100 μg/mL of TBE-50 or TBE-70 for 2, 5, or 7 days (d2 to d9) as shown in Figure 5a. The intracellular lipid content was measured by Oil Red O staining. On Day 7 (d9), change of adipocyte differentiation was presented with Oil Red O staining (Figure 5b). After 7 days of incubation, TBE-70 inhibited the intracellular lipid content by 15.2% compared to the control cells, whereas TBE-50 did not result in significant change (Figure 5c). To determine the intracellular TG content, 3T3-L1 adipocytes were treated with 0 (control), 1, 50, and 100 μg/mL of TBE-50 and TBE-70 for 7 days. The 100 μg/mL of TBE-70 significantly decreased the TG content by 22.3% compared to the control cells. Further, the intracellular TG content of the TBE-70 was found to be lower than that of TBE-50 by 15.3% (Figure 5d). In a previous study, supplementation of buckwheat leaf and flower mixture resulted in reduced weight gain and plasma lipid concentrations in rats fed a high-fat diet [28]. In addition, a study in humans revealed that the rutin-rich tartary buckwheat showed potential effects on decreasing the body fat percentage and body weight in adults [13]. Meanwhile, the anti-adipogenic effects of phenolic acids present in tartary buckwheat such as rutin [29], ferulic acid [30], and quercetin [31,32,33] have been reported. Our results showed that TBEs reduced the intracellular lipid and TG concentrations during adipocyte differentiation. These results imply that TBEs would be beneficial at suppressing fat accumulation, and these effects seem to be a result of complex action of phenolic acids and rutin in TBEs.

### 2.4. Effect of TBEs on GPDH Activity in Adipocytes

**Figure 6:**
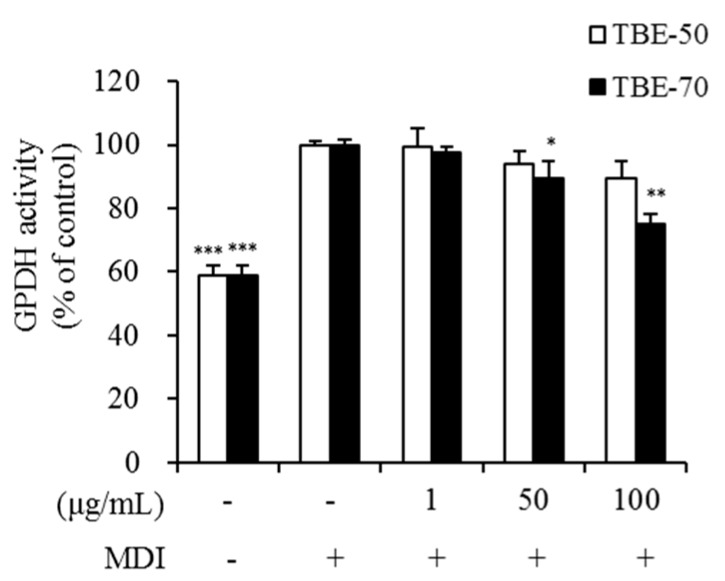
Effects of TBEs on GPDH activity in adipocytes. GPDH activity was determined using a GPDH assay kit. 3T3-L1 adipocytes were treated with 0 (MDI treated control), 1, 50, and 100 µg/mL of TBE-50 and TBE-70, and incubated for 7 days. MDI, medium containing 3-isobutyl-1-methylxanthine, dexamethasone, and insulin. Values are expressed as mean ± SE (*n* = 3) of three independent experiments. * *p* < 0.05, ** *p* < 0.01 and *** *p* < 0.001 vs. MDI-treated control.

One of the possible methods of inhibiting lipid accumulation in adipocytes is blocking lipid synthetic pathways in their cells. The enzyme glycerol-3-phosphate dehydrogenase (GPDH) plays a major role in the TG synthesis pathway and is linked to characteristic changes that occur during adipose conversion [34]. To elucidate the mechanism by which TBEs inhibits lipid accumulation, GPDH activity was measured in 3T3-L1 adipocytes. The 3T3-L1 adipocytes were incubated with 0 (control), 1, 50, and 100 μg/mL of TBE-50 and TBE-70, and incubated for 7 days. GPDH activity was significantly decreased by 10.7% and 25.1%, respectively, in the presence of 50 and 100 μg/mL of TBE-70, compared to the untreated control (Figure 6). However, no significant differences in GPDH activity were observed in the presence of TBE-50. Yoon and others [19] have reported that an 80% ethanolic extract of tartary buckwheat showed relatively higher inhibition of GPDH activity and lipid accumulation than the common buckwheat extract in 3T3-L1 adipocytes. In addition, Hsu and Yen have reported that rutin inhibits intracellular TG accumulation and GPDH activity in adipocytes during differentiation [29]. Similarly, in our result, GPDH activity was dose-dependently reduced by the TBE-70 in 3T3-L1 adipocytes. Thus, it can be speculated that TBEs may have a suppressive effect on lipid synthesis partially via reduction of GPDH activity during adipocyte differentiation.

### 2.5. Effect of TBEs on mRNA Expression of Genes Involved in Fatty Acid Synthesis in Adipocytes 

During adipogenesis, enhanced expression of c-fos, c-jun, junB, c-myc, and CCAAT/enhancer binding proteins (C/EBP)-β and -δ is observed [35]. Activated C/EBP-β and -δ mediate the expression of peroxisome proliferator-activated receptor γ (PPARγ) and C/EBP-α [35]. To understand the mechanism underlying the anti-adipogenic effect of TBEs in adipocyte, the mRNA levels of genes involved in fatty acid synthesis were measured. At the molecular level, adipogenesis is driven by a complex signaling cascade that involves key transcription factors, such as proliferator-activated receptor-γ (PPAR-γ) and CCAAT/enhancer binding protein-α (CEBP-α) [36]. When PPAR-γ expression is stimulated, lipid biosynthesis pathways are activated through the expression of target genes, such as CEBP-α and aP2 [37,38]. The adipogenic marker gene adipocyte protein 2 (aP2) is highly expressed as a result of adipocyte differentiation [39]. Acetyl-CoA carboxylase (ACC) is the rate-limiting enzyme of fatty acid synthesis that catalyzes the carboxylation of acetyl-CoA to produce malonyl-CoA [40]. Fatty acids are synthesized from malonyl-CoA through processes catalyzed by fatty acid synthase (FAS) and stearoylcoenzyme A desaturase-1 (SCD-1) [41]. Suppression of genes involved in fatty acid synthesis, such as ACC, FAS, and SCD-1, leads to reduction of adipocyte triglyceride synthesis and accumulation [42]. In this study, we investigated the mRNA levels of genes involved in fatty acid synthesis, such as PPAR-γ, CEBP-α, aP2, ACC, FAS, and SCD-1 in 3T3-L1 adipocytes to investigate the mechanisms involved in the anti-adipogenic effect of TBEs. The 3T3-L1 adipocytes were treated with 100 µg/mL of TBE-50 and TBE-70 and incubated for 7 days. The mRNA levels of PPAR-γ, CEBP-α, aP2, ACC, FAS, and SCD-1 were significantly reduced by 39.3%, 52.0%, 31.3%, 44.0%, 34.7%, and 35.0%, respectively, in TBE-70 compared to untreated control cells (Table 1). TBE-50 significantly decreased the mRNA level of CEBP-α by 23% compared to the untreated control. Additionally, the mRNA level of CEBP-α, ACC, FAS, and SCD-1 was significantly lower (by 37.3%, 36.6%, 31.9%, and 32.9%, respectively) in the TBE-70 than TBE-50. In a previous study, it has been reported that oral administration of germinated buckwheat diminished fatty liver by suppressing the expression of key adipogenic transcriptional factors, such as PPAR-γ and CEBP-α in hepatocytes [43]. In addition, treatment of phenolic acids including rutin downregulates mRNA expression of PPAR-γ, C/EBP-α, and leptin, as well as upregulates adiponectin mRNA expression in differentiating adipocytes [29]. Our results indicate that the mRNA levels of PPAR-γ, CEBP-α, aP2, ACC, FAS, and SCD-1 decreased in cells treated with TBE-70 more so than those in cells treated with TBE-50. In contrast, TBE-50 only showed downregulation of CEBP-α expression. Therefore, the results indicate that TBEs may have anti-adipogenic effects in adipocytes, and the inhibitory effect could be in part explained by suppression of adipocyte-specific gene expression including PPAR-γ, CEBP-α, aP2, ACC, FAS, and SCD-1.

### 2.6. Effect of TBEs on mRNA Expression of Inflammatory Mediators and NO Production in Adipocytes 

The tumor necrosis factor-α (TNF-α), interleukin-6 (IL-6), monocyte chemoattractant protein 1 (MCP-1), and inducible nitric oxide synthase (iNOS) are involved in the derivation and maintenance of chronic inflammatory responses in obesity [44,45]. In our study, to elucidate the inflammatory response of TBE, we analyzed the mRNA expression of inflammatory mediators such as TNF-α, IL-6, MCP-1, and iNOS, and nitric oxide (NO) production in adipocytes. As the lipid content in the adipose tissue increases, adipocytes synthesize TNF-α and IL-6, thus directly causing the inflammatory response [46]. Toll-like receptors (TLRs) are a family of molecules that are involved in the innate immunity. In obese adipocytes, the expression of several TLRs is elevated and activation of these receptors is thought to produce chemotactic signals, such as MCP-1, which provoke macrophage infiltration [47,48]. Activated macrophages in adipose tissue secrete pro-inflammatory cytokines, which leads to chronic low-grade inflammation in obesity [47]. It has been reported that treatment of citrus flavonoid naringenin during adipocyte differentiation inhibits TLR2 expression [48]. In addition, naringenin treatment suppresses NO production and TNF-α secretion from RAW 264.7 macrophages [49]. Moreover, coculture of 3T3-L1 adipocytes and RAW 264.7 macrophages enhances the production of TNF-α, MCP-1, and NO compared with the control cultures, while the treatment with naringenin chalcone dose-dependently inhibits the production of these pro-inflammatory mediators [50]. 

**Figure 7:**
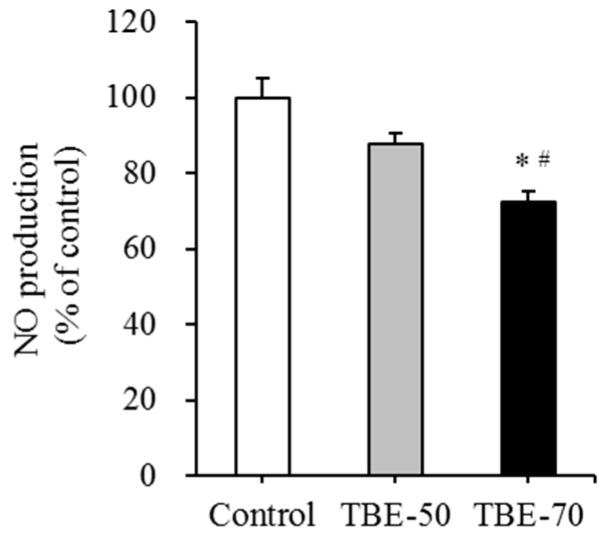
Effects of TBEs on NO production in adipocytes. 3T3-L1 adipocytes were treated with 100 µg/mL of TBE-50 or TBE-70, and incubated for 7 days. The production of NO was measured using Griess reagent. Values are expressed as mean ± SE (*n* = 3) of three independent experiments. * *p* < 0.05 vs. control. ^#^ *p* < 0.05 vs. TBE-50.

In this study, we investigated the mRNA expression of inflammatory mediators, such as TNF-α, IL-6, MCP-1, and iNOS, and NO production on TBE-50 and TBE-70 in 3T3-L1 cells. The cells were treated with 100 µg/mL of TBE-50 and TBE-70 and incubated for 7 days. TBE-70 decreased the mRNA levels of inflammatory mediators such as TNF-α, IL-6, MCP-1, and iNOS by 52.8%, 48.7%, 36.3% and 59.7%, respectively, compared with those in the untreated control (Table 2). TBE-50 decreased the mRNA level of iNOS by 35.7% compared to the untreated control. Further, the mRNA level of IL-6 and iNOS was significantly lower (by 32.5% and 37.3%, respectively) in the TBE-70 than TBE-50. NO production was significantly inhibited by 27.7% in TBE-70 compared to the control, and the TBE-70 was decreased more than TBE-50 (by 15.5%) (Figure 7). It has been reported that iNOS is a key mediator in obesity-induced inflammation, and an enzyme involved in the production of NO [51]. In a previous study, tartary buckwheat fractions and rutin effectively inhibited the production of ROS, NO, and IL-6, and downregulated the mRNA expression levels of pro-inflammatory factors including nuclear factor kappa B, cyclooxygenase-2, and iNOS in lipopolysaccharide (LPS)- and interferon-γ-stimulated RAW 264.7 cells [15]. In particular, rutin exhibits anti-inflammatory properties by inhibiting the release of TNF-α from monocytes [52] and human peripheral blood neutrophils [53]. Moreover, rutin exerts protective effects on inflammatory diseases such as acute pancreatitis [54], diabetic cardiomyopathy [55], neuroinflammation [56] in vivo. Meanwhile, phenolic acid present in tartary buckwheat-such as protocatechuic acid [57,58], syringic acid [59], ferulic acid [58], sinapic acid [60], quercetin [61], and rutin [62]-have shown beneficial effects on LPS-induced inflammation in RAW 264.7 cells have been reported, indicating that rutin and other phenolic acids present in TBEs might be multiply associated with anti-inflammatory effects of TBEs in adipocytes. In the present study, we first found that TBEs decreased the mRNA levels of inflammatory mediators, such as TNF-α, IL-6, MCP-1, and iNOS, and NO production during adipocyte differentiation, and these results suggest that TBEs would be beneficial in suppressing obesity-related inflammation.

## 3. Materials and Methods 

### 3.1. Materials

The 3T3-L1 cells were obtained from American Type Culture Collection (Manassas, VA, USA). Dulbecco’s modified Eagle’s medium (DMEM), glutamine, penicillin-streptomycin, fetal bovine serum (FBS), and TRIzol reagent were obtained from Invitrogen (Carlsbad, CA, USA). The cell count kit-8 (CCK-8) was purchased from Dojindo Laboratories (Kumamoto, Japan). An assay kit for TG was obtained from Asan Pharmaceutical Co. (Seoul, Korea). The GPDH activity assay kit was from Takara (Kyoto, Japan). The bicinchoninic acid (BCA) protein assay kit was obtained from Thermo Scientific (Pittsburgh, PA, USA). Universal SYBR^®^ Green PCR Master Mix was purchased from Qiagen (Chatsworth, CA, USA). M-MLV reverse transcriptase was purchased from Promega (Madison, WI, USA). The NO assay kit was purchased from Thermo Scientific (Pittsburgh, PA, USA).

### 3.2. Preparation of Tartary Buckwheat Extracts

The extracts of tartary buckwheat were kindly supplied by the SKBioland Co. (Ansan, Gyeonggi, Korea). Tartary buckwheat was purchased from buckwheat associative corporation in Jeju, Korea. The extraction was performed as follows: 100 g of tartary buckwheat was subjected to extraction with 50% or 70% ethanol (1:15 (*w*/*v*)) at 80 °C for 3 h, and was then filtered. The filtrate was vacuum evaporated and the concentrated liquid was spray-dried (Dongjin ENG Inc., Siheung, Korea) and used as TBE-50 and TBE-70, respectively.

### 3.3. HPLC Analysis

The rutin content of TBE-50 and TBE-70 was determined by HPLC. Briefly, each sample was dissolved in methanol in 1 mg/mL concentration and was filtered through a 0.45 μm PVDF syringe filter. The Agilent technologies 1260 infinity (Agilent Technologies, Santa Clara, CA, USA) with a ZORBAX Eclipse XDB-C18 column (250 mm × 4 mm, 5 μm pore size, Agilent technologies) was used for analysis. The mobile phases consisted of 0.1% formic acid in water (Solvent A) and 0.1% formic acid in acetonitrile (Solvent B). The gradient elution used was as follows: 0–5 min, 5% B, 5–30 min, 10% B; 30–40 min, 40% B; 40–45 min, 90% B; 45–55 min, 5% B. The flow rate was 0.3 mL/min and the volume of sample injection was 20 μL. The column temperature was constant at 30 °C.

### 3.4. Cell Culture

The culture of 3T3-L1 cells was initially conducted in DMEM containing 10% (*v*/*v*) FBS, 100 units/mL penicillin, 100 μg/mL streptomycin, and 2 mM glutamine under conditions of 37 °C and 5% CO_2_. For the induction of adipocyte differentiation, the preadipocytes were cultured to confluence (Day 0, d0) and exposed to a differentiation medium containing 0.5 mM isobutylmethylxanthine, 1 μM dexamethasone, and 5 μg/mL insulin (MDI) for 2 days (d2). The cells were cultured with 5 μg/mL insulin for 2 days (d4) and then subsequently cultured in DMEM containing 10% FBS for 5 days (d9). TBE-50 and TBE-70 were treated with the medium for 7 days (from d2 to d9). Cells incubated without treatment served as a control. All assays were carried out in triplicate. 

### 3.5. Cell Viability Assay 

The cytotoxicity of TBE-50 and TBE-70 in adipocytes was evaluated by the WST-8 [2-(2-methoxy-4-nitropheyl)-3-(4-nitrophenyl)-5-(2,4-dinitrophenyl)-2H-tetrazolium, monosodium salt method, using a commercial CCK-8 kit as described previously [63]. Differentiated 3T3-L1 adipocytes were incubated for 1, 2, 5, or 7 days with TBE-50 or TBE-70 (at a dose of 0, 0.1, 1, 10, 50, 100, or 500 µg/mL). The absorbance was read using a Varioskan plate reader (Thermo Electron, Waltham, MA, USA) at 450 nm, and the results are expressed as the percentage of the control. All assays were carried out in triplicate.

### 3.6. Oil-Red O Staining

Lipid content was measured as previously described [63]. Briefly, 3T3-L1 adipocytes were treated with 100 μg/mL of TBE-50 or TBE-70. On Days 2, 5, and 7, the cells were rinsed in phosphate-buffered saline (PBS) (pH 7.4) and then fixed in 10% (*v*/*v*) formalin in PBS. To quantify accumulated lipid levels, cells were stained for 15 min with oil-red O dye (6 parts of saturated oil-red O in isopropanol and 4 parts of water). Oil droplets stained with oil-red O were dissolved in 4% (*v*/*v*) Nonidet P-40 in isopropanol and quantified by measuring absorbance at 520 nm [64]. The results are expressed as the percentage of the control.

### 3.7. TG Assay

For the measurement of intracellular TG, a colorimetric TG Assay kit was used according to the method as described previously [63]. 3T3-L1 adipocytes were lysed using a buffer containing 1% Triton X-100 in PBS, and the level of TG was determined using a commercial TG assay kit. Cellular TG content was then normalized to the protein concentration measured by a BCA protein assay kit.

### 3.8. GPDH Activity

GPDH activity was analyzed as described previously [65] using a commercial kit. In brief, 3T3-L1 adipocytes were treated with 100 μg/mL of TBE-50 or TBE-70. The cells were then disrupted by homogenization and centrifuged at 4 °C for 10 min. The supernatant was assayed for the GPDH activity by monitoring the decrease of NADH in the presence of dihydroxyacetone phosphate and measuring absorbance at 340 nm. Cellular protein level was determined using a BCA protein assay kit. The results are expressed as the percentage of the control.

### 3.9. Real-Time qPCR

Total RNA was extracted from 3T3-L1 adipocytes by using TRIzol Reagent. The cDNAs were synthesized from 4 μg RNA by using M-MLV reverse transcriptase. Quantitative real-time PCR was then carried out in 25 μL of Universal SYBR^®^ Green PCR Master Mix using a fluorometric thermal cycler (Rotor-GeneTM 2000; Corbett Research, Mortlake, NSW, Australia). Primer3 software (Version 2.3.6, MA, USA) was used for the primer design [66]. The sequences of the primers used are presented in Table 3. For relative quantification, the delta–delta Ct method was used [67], and β-actin was used as an endogenous control. Values presented represent fold changes compared to the control.

### 3.10. NO Production

The production of NO was measured as nitrite using a commercial kit according to the manufacturer’s instructions. 3T3-L1 adipocytes were treated with 100 μg/mL of TBE-50 or TBE-70 for 7 days. The NO released into the medium was reacted with Griess reagent (1% sulfanilamide +0.1% naphthylendiamine dihydrochloride, 1:1) for 30 min at room temperature, and the absorbance was measured at 548 nm. The nitrite concentration was determined using sodium nitrite as a standard. The cellular protein concentration was determined using a BCA protein assay kit. The NO concentrations were normalized to the cellular protein content. Values are presented as percentage of untreated control.

### 3.11. Statistical Analysis 

Values are expressed as mean ± standard error of the mean (SEM). Statistical analyses were performed using SPSS software (version 23; IBM Corporation, Armonk, NY, USA). The significance of differences between two groups of TBE-50 and TBE-70 with the same concentration was determined with a Student’s t-test (two-tailed). Significant differences among different concentrations of treatment group were analyzed using a one-way analysis of variance (ANOVA), followed by Tukey’s multiple comparison tests. *p* < 0.05 indicated a significant difference.

## 4. Conclusions

Taken together, our results demonstrate that TBEs may inhibit adipogenesis and inflammatory response during adipocyte differentiation of 3T3-L1 cells. These effects of TBEs were partially mediated by reducing GPDH activity and NO production, and by modulating the expression of genes involved in fatty acid synthesis and inflammatory mediators. Thus, TBEs may be useful as a potential food ingredient to prevent obesity-associated inflammation.

## Figures and Tables

**Table 1:** Effects of TBEs on mRNA expression of adipocytes-specific genes in adipocytes.

Genes	Control	TBE-50	TBE-70
PPAR-γ	1.00 ± 0.06	0.85 ± 0.13	0.61 ± 0.06 *
CEBP-α	1.00 ± 0.07	0.77 ± 0.03 *	0.48 ± 0.04 **^,#^
aP2	1.00 ± 0.02	0.86 ± 0.10	0.69 ± 0.01 *
ACC	1.00 ± 0.12	0.88 ± 0.09	0.56 ± 0.03 *^,#^
FAS	1.00 ± 0.12	0.88 ± 0.09	0.56 ± 0.03 *^,#^
SCD-1	1.00 ± 0.03	0.97 ± 0.10	0.65 ± 0.04 *^,#^

mRNA level was measured using real-time quantitative polymerase chain reaction (qPCR). Values represent fold changes compared to the control. Data are expressed as mean ± SE of at least three independent experiments, each performed in triplicate (*n* = 3). * *p* < 0.05 and ** *p* < 0.01 vs. control. ^#^ *p* < 0.05 vs. TBE-50.

**Table 2:** Effects of TBEs on mRNA expression of inflammation mediators in adipocytes.

Genes	Control	TBE-50	TBE-70
TNF-α	1.00 ± 0.03	0.71 ± 0.06	0.47 ± 0.11 **
IL-6	1.00 ± 0.09	0.76 ± 0.08	0.51 ± 0.04 **^,#^
MCP-1	1.00 ± 0.06	0.80 ± 0.06	0.64 ± 0.06 *
iNOS	1.00 ± 0.10	0.64 ± 0.04 *	0.40 ± 0.03 **^,#^

mRNA level was measured using qPCR. Values represent fold changes compared to the control. Data are expressed as mean ± SE of at least three independent experiments, each performed in triplicate (*n* = 3). * *p* < 0.05 and ** *p* < 0.01 vs. control. ^#^ *p* < 0.05 vs. TBE-50.

**Table 3:** Primers used for qPCR.

Name	GeneBank No.	Primer Sequence (5′-3′)
ACC	AY451393	F: CAAGTGCTCAAGTTTGGCGC
		R: CAAGAACCACCCCGAAGCTC
aP2	NM_024406	F: CGACAGGAAGGTGAAGAGCA
		R: ATTCCACCACCAGCTTGTCA
β-actin	NM_007393	F: GGACCTGACAGACTACCTCA
		R: GTTGCCAATAGTGATGACCT
CEBP-α	NM_007678	F: ATAGACATCAGCGCCTACAT
		R: TCCCGGGTAGTCAAAGTCAC
FAS	AF127033	F: CTGGCATTCGTGATGGAGTC
		R: TGTTTCCCCTGAGCCATGTA
IL-6	NM_031168	F: CCTTCCTACCCCAATTTCCA
		R: TAACGCACTAGGTTTGCCGA
iNOS	BC062378.1	F: GCTACTGGGTCAAAGACAAG
		R: GCTGAACTTCCAGTCATTGT
MCP-1	NM_019812	F: TGCTGACCCCAAGAAGGAAT
		R: TGAGGTGGTTGTGGAAAAGG
PPAR-γ	NM_011146	F: TTGATTTCTCCAGCATTTCT
		R: TGTTGTAGAGCTGGGTCTTT
SCD-1	AF509567	F: ATGGATATCGCCCCTACGAC
		R: GATGTGCCAGCGGTACTCAC
TNF-α	NM_013693	F: AGCACAGAAAGCATGATCCG
		R: CCACAAGCAGGAATGAGAA
